# The hematopoietic master regulator RUNX1 reshapes the epigenetic landscape at the onset of hematopoiesis

**DOI:** 10.1186/1756-8935-6-S1-O18

**Published:** 2013-03-18

**Authors:** Monika Lichtinger, Nadine Obier, Richard Ingram, Rebecca Hannah, Maarten Hoogenkamp, MS Vijayabaskar, Mengchu Wu, Salam A Assi, Daniel G Tenen, David R Westhead, Valerie Kouskoff, Georges Lacaud, Berthold Göttgens, Constanze Bonifer

**Affiliations:** 1 Section of Experimental Haematology Leeds Institute of Molecular Medicine, University of Leeds, Leeds LS9 7TF, UK; 2 School of Cancer Sciences, University of Birmingham, Birmingham B152TT, UK; 3 Cambridge Institute of Molecular Medicine, Cambridge CB2 0XY, UK; 4 Paterson Institute for Cancer Research, University of Manchester, Manchester M20 4BX, UK; 5 Cancer Science Institute, National University of Singapore, Republic of Singapore; 6 Harvard Stem Cell Institute, Harvard Medical School, Boston, MA, USA; 7 Faculty of Biological Sciences, University of Leeds, Leeds LS2 9JT, UK

## 

Hematopoiesis in the embryo originates from mesodermal cells and proceeds via a common precursor (hemangioblast) of endothelial cells and blood cells. Hemangioblasts give rise to specialised endothelial cells (hemogenic endothelium) which subsequently undergo a transition into hematopoietic precursor cells. These cell fate decisions are governed by lineage-specific transcription factors, such as RUNX1, SCL/TAL1, FLI-1, PU.1 and C/EBP family members. In our work we study how dynamic shifts in the transcriptional regulatory network during this developmental pathway are regulated and how transcription factors control the activation of hematopoietic genes. We are also investigating, how these factors interact with each other and with the chromatin landscape.

To this end, we measured the genome-wide dynamics of chromatin alterations during the different steps of formation of the hematopoietic system from mesodermal cells using ES cell differentiation as model. We show that the hematopoietic program is already primed in the hemogenic endothelium as indicated by the binding of SCL/TAL1, FLI-1, C/EBPβ and enhancer-bound RNA-Polymerase II as well as the appearance of DNAsel hypersensitive sites. RUNX1 is absolutely required for the transition from hemogenic endothelium cells into hematopoietic progenitors. To obtain mechanistic information how this factor drives this process, we examined the assembly of hematopoietic transcription factors on their targets before and after this transition. Using an inducible system, we show that after induction RUNX1 binds to primed, but also novel elements and increases their histone acetylation. Moreover, RUNX1 initiates rapid global alterations in the binding patterns of SCL/TAL1 and FLI1, involving both the extinction of binding sites as well as the establishment of new sites. A significant fraction of new elements bind SCL/TAL1 and FLI1 in close proximity to RUNX1 in a pattern that is specific for hematopoietic cells.

RUNX1 has previously shown to be expendable in hematopoietic precursor cells once they have formed from the hemogenic endothelium. By precisely timed withdrawal studies we show that immediately after RUNX1 induction altered transcription factor complex assembly at many genes is still reversible, but not at all of them. Our experiments suggest a dynamic interplay between RUNX1 and other transcription factors that dictates the half-life of factor assemblies and their dependency on RUNX1 and give a fascinating insight into how a single master regulator shapes the epigenetic landscape.

